# Discomfort and distress in slum rehabilitation: Investigating a rebound phenomenon using a backcasting approach

**DOI:** 10.1016/j.habitatint.2019.03.010

**Published:** 2019-05

**Authors:** Ramit Debnath, Ronita Bardhan, Minna Sunikka-Blank

**Affiliations:** a Behaviour and Building Performance Group, Department of Architecture, University of Cambridge, CB21PX, UK; b Centre for Sustainable Development, Department of Engineering, University of Cambridge, CB21PZ, UK; c Centre for Research in Arts, Social Science and Humanities, University of Cambridge, CB39DT, UK; d Centre for Urban Science and Engineering, Indian Institute of Technology Bombay, 400076, India

**Keywords:** Slum rehabilitation, Sustainability, Homeostasis, Discomfort, Distress, Urban, Poverty, Energy, India

## Abstract

Slum rehabilitation policies in India is observed to have a rebound effect on the occupants, where rehabilitated occupants move back to the horizontal slums. In this study, we investigate the cause behind this rebound phenomenon based on a theory of homeostasis, where the loss of homeostasis refers to occupants' heightened discomfort and distress in their built environment. A novel methodological framework was developed to investigate it based on the principles of participatory backcasting approach and the theory of homeostasis. Thirty households in Mumbai's slum rehabilitation housing were interviewed to determine the social, economic and environmental cause of distress and discomfort. Granular information was obtained by further investigating the factors that influence occupants' attitude, emotions, health, control and habits in their built environment that regulates their holistic comfort and lack of stress. The causal linkages among these factors were established using a qualitative fault tree. Results show two primary cause of distress and discomfort in the study area owing to economic distress and built environment related discomfort. Economic distress was from low-income and high electricity bills due to higher household appliance ownership, and built environment discomfort was due to lack of social spaces and poor design of the slum rehabilitation housing. This study showed that mitigating such non-income drivers of distress and discomfort can prevent rebound phenomenon and improve the sustainability of the slum rehabilitation process.

## 1 Introduction

United Nations Human Settlement Program had estimated that China (180 million) and India (104 million) had the higher number of slum dwellers in the world in 2012, and these numbers are set to increase in the next decade owing to their urbanisation trend (UN-Habitat, 2014). Between 1950 and 2005, China urbanised at a rate of 41 percent, compared to 29 percent in India (Dobbs & Sankhe, 2010). However, the urban population living in slums in both the countries remained comparable in 2010 (approximately 28.2 percent of the urban population lived in slums in 2010 in India and China) (UN Habitat, 2010). It is due to distinct macro and micro level forces behind India's urbanisation trends. Macro approaches, i.e., studying national aggregated trends in association with macroeconomic development, clarify the speed of urban growth and urban structural conditions. Micro approaches are needed to understand the city from the perspective of citizens and evolving urban cultures (Nijman, 2015). The microanalysis indicates the persistence and growth of slums due to India's lethargic formal manufacturing sector that fuels small-scale manufacturing (Nijman, 2015). It leads to large-scale rural-urban migration to megacities like Mumbai, Delhi, Bengaluru, Chennai and Kolkata and is a significant force behind the rapid urban slum formation in India (Bardhan, Sarkar, Jana, & Velaga, 2015a).

It is well understood in the policy sphere that the slum life has a significant adverse effect on health, education, labour and women. It is also widely discussed in the current policy discourses that improving the living conditions of the slums must include concerned-efforts of all the stakeholders (Nijman, 2015). Policies should reassure the good-will of authorities, the engagement of the concerned communities and the better understanding of urban poverty by the general public (UN-Habitat, 2016, pp. 1–96). However, despite progressive policies aimed at slum eradication, urban slums in India have expanded, and their population have increased (Nijman, 2015). It is due to primarily two reasons, first is India's lethargic manufacturing sector that impedes rapid urbanisation while concurrently fuelling small-scale and informal manufacturing in urban environments which attracts a low-skilled and low-wage workforce from rural areas (Nijman, 2015). The second cause is due to the inherent weakness of Indian planning and planning institutions that cause disjointedness to the intention of slums upgradation and eradication (Roy, 2009). In the current policy discourse of affordable housing for the low-income population, the Slum Rehabilitation Scheme (SRS) is a widely adopted policy instrument due to its market-driven attributes (discussed in detail in Section 2.1).

In Mumbai, slum rehabilitation is performed through a state-operated agency called the Slum Rehabilitation Authority (SRA). It is responsible for the evaluation and approval of slum redevelopment proposals submitted by developers. In the entire rehabilitation process, the developers have full discretion on the quality of rehabilitated buildings, which affects the living standards, so much so that, these rehabilitation buildings are transformed into ‘vertical slums’ (Zhang, 2016), (Debnath, Bardhan, & Jain, 2017). It forces the occupants to abandon these houses and either move back to the horizontal slums or create an informal settlement in some other parts of the city. We termed this a ‘*rebound phenomenon*’ of this slum rehabilitation process. It might seem that poverty is the primary cause of the rebound behaviour as the occupants cannot cope-up with the increased cost of living and low-income, as suggested by Restrepo (Restrepo, 2010). However, it is the added burden of transition or ‘changing institutions’ that pushes them into a vicious cycle of poverty even after moving into a permanent structure (Nijman, 2008). We argue that this burden comprises of broader economic, social and environmental elements that affect occupants' attitudes and behaviour which adversely affects their well-being by causing discomfort and distress in their newly built environment (i.e. in the slum rehabilitation housing).

While the actual cause of this rebound phenomenon is yet unknown, it is often discussed in the literature that the horizontal slums help to maintain the informal economy which creates job opportunities for the occupants. Furthermore, the design of the current rehabilitation housing restricts them from accessing communal spaces which are an integral part of their lives in slums. Occupants' discomfort is further exaggerated by the poor quality of the housing units, which are often characterised by the lack of ventilation, thermal discomfort, lack of daylight and poor indoor air quality in the living spaces (Nijman, 2015), (Bardhan, Debnath, Malik, & Sarkar, 2018b), (Yardley, 2010). In economic and planning theory, such a rebound effect in real-estate dynamics is often discussed using resident mobility theory (Turner, 1968) which aid in accurately measuring the impact of a policy. Restrepo (Restrepo, 2010) investigated similar rebound phenomenon using resident mobility as an indicator to investigate the effect of slum rehabilitation policy on the slum dwellers. It was found that the rebound may be caused by a significant mismatch between household needs and built environment design; and incompatibility between the higher cost of living and household's economic status (Restrepo, 2010). These studies establish the importance of income-based factors that cause significant distress on the occupants' well-being living in the slum rehabilitation housing. However, they do not account for the social and environmental dimensions of occupants' distress and discomfort that also significantly affects the overall quality of life of the occupants living in these areas (Bardhan, Debnath, Jana, & Norford, 2018c), (Vaid & Evans, 2017).

Here, we make a generalised assumption that the rebound phenomenon is caused due to occupants' economic, social and environmental discomfort and distress in their built environment. While low-income remains a constant source of distress and distress, we investigate the influence of non-income factors that contribute to it. The non-income factors comprises of physical, social, behavioural and psychological elements that regulate the attitudes, habits and emotions of the occupants towards maintaining a balanced state of comfort and lack of stress in their built environment, as per Ortiz et al.‘s theory of homeostasis (Ortiz, Kurvers, & Bluyssen, 2017) (discussed in detail in Section 2.2). This theory provides a robust background to this research as it combines these non-income drivers of comfort and lack of stress to explain the contextual nature of occupants' health and well-being (Ortiz et al., 2017).

We extend this theory of homeostasis (Ortiz et al., 2017) by developing a novel conceptual framework for evaluating the causes behind occupants' discomfort and distress from an individualistic scale to a neighbourhood scale (detail are in Section 3). We hypothesise that the rebound phenomenon in the slum rehabilitation housing in Mumbai can be prevented by mitigating occupants' discomfort and distress in their built environment which is driven by the non-income factors. To test this hypothesis, we address two key research questions, first, ‘*What causes discomfort and distress in the slum rehabilitation housing?*’, and the second, ‘*How can the community help in reducing their discomfort and distress?*’.

To address these research questions, we designed an innovative methodology to derive a proof-of-concept to support our assumptions regarding the non-income drivers of the rebound phenomenon. It consisted of designing a conceptual framework that can logically accommodate the non-income drivers of discomfort and distress based on the theory of homeostasis of Ortiz et al. (Ortiz et al., 2017) (see section 3). It was utilised in designing the participatory surveys conducted in a slum rehabilitation housing society in Mumbai, called Natvar Parekh Complex (NPC). These surveys were designed using a participatory backcasting approach to generate desired and undesired scenarios to enumerate occupants’ perception of discomfort and distress in the study area. Backcasting-based surveys were thoroughly conducted as per the guidelines of Robinson J. (Robinson, 2003).

Backcasting approach is ‘working backwards’ from a future end-point to the present, and is widely used in policy modelling (Robinson, 1982). Our methodological innovation lies in customising this policy modelling technique in the investigation of the multi-dimensional causes of occupants' discomfort and distress in low-income housing. The mode of surveys were unstructured interviews, and when it was combined with the participatory backcasting method, it gave a multi-perspective bandwidth to the interviewers to understand the current cause of discomfort and distress, and the potential solutions to mitigate them to reduce the rebound phenomenon. It is discussed in detail in section 4. Lastly, the survey results were analysed using a qualitative Fault Tree Analysis (FTA) that listed the causal linkages of occupants' discomfort and distress in the study area and generated the proof-of-concept of the proposed theoretical framework for examining the actual causes of rebound phenomenon across the economic, social and environmental dimensions of occupants' well-being. The inclusion of these non-income drivers in the current slum rehabilitation policy-making process is expected to improve the sustainability of the SRS scheme and reduce its rebound effect.

## 2 Background

### 2.1 State of slum rehabilitation housing in Mumbai

The urbanisation process in Mumbai has been coherent with the housing crisis and urban informality for over 150 years. At present, more than 50% of the population lives in slum areas (Bardhan et al., 2015a). The proliferation of urban slums in Mumbai have been consistent since the independence of India in 1947 despite progressive policies aimed at their removal or rehabilitation. The literature points out several explanations, of which most of the studies focus on spatiotemporal identification and its spatial spread concerning the real-estate undercurrents. However, understanding the policy impacts on reduction and rehabilitation of slums are less studied (Nijman, 2015), (Bardhan et al., 2015a), (Bardhan et al., 2018c), (Birch, Chattaraj, andWachter, Eds., & Slums, 2016), (Nolan, 2015). Macroeconomic explanation of the sprawling of slums in big cities commonly states that urban slums function as cheap labour. Roy (Roy, 2009) provides a compelling argument stating that the very informality of Indian planning and planning institutions causes incoherence to the intention to fix the problem of slums, despite the consistent effort of the federal government. It leaves the slum rehabilitation and urban renewal policies a plain ‘board-room’ mandates, which lacks the structural forces to strengthen the micro-level (informal) institutions of the slum dwellers that shape their built environment along with their mindset and aspirations (Bardhan et al., 2018b).

Slum rehabilitation and upgradation programs have always been a primary political agenda of the Government of India. These programs are designed in a top-down manner to construct public housing that would improve housing standards and close the housing deficit. However, little is known about the *after-effects* of these policies on the occupants’ overall well-being. Market forces regarding land and finance drive the entire slum rehabilitation process that creates intense competition for the slumlands among the private developers (Nijman, 2008). These developers promise to construct free slum rehabilitation houses (SRH) by incentivising the floor space index (FSI). It provides them with the more significant advantage of building taller SRH in a smaller portion of the area while maximising occupancy. At the same time, constructing luxury apartment in the rest of the land and selling them in the premium real-estate market of Mumbai. It makes the rehabilitation process entirely market-driven, resulting in sub-standard housing design leading to poor quality of life (Bardhan et al., 2015a), (Bardhan, Debnath, Malik, & Sarkar, 2018d) (see appendix for the current slum rehabilitation process).

Recent research on the quality of slum rehabilitation housing (SRH) has revealed that occupants tend to seek more healthcare visits due to lack of ventilation and fresh air exchanges (Bardhan et al., 2018c). Thermal comfort levels were also found to decrease in vertical structures as compared to the horizontal slums, and it is regarded as a significant concern for the occupants (Nutkiewicz, Jain, & Bardhan, 2018). A study by Bardhan et al. (Bardhan et al., 2018d) has shown that social and architectural elements influence occupancy in the SRH, such that occupants prefer horizontal slums due to ample social spaces. It also adds to their overall comfort in their built environment (Debnath et al., 2017). A behavioural study by Vaid and Evans (Vaid & Evans, 2017) have shown that on moving to formal housing (like the SRH), the slum dwellers felt satisfied and contented with their aspirations. However, owing to poor design and lack of social spaces, their perception of the SRH scheme changes as they move on with their daily life. They feel significant improvements are needed regarding hygiene, cleanliness, safety, comfort levels and indoor air quality. The occupants tend to rely on the local government for their ‘holistic comfort’ in their built environment. We explore this notion of ‘holistic comfort’ through the theory of homeostasis, discussed in section 2.2.

### 2.2 Rebound phenomenon and the theory of homeostasis

The slum rehabilitation process is an institutional change that involves shifting of an entire informal built environment to a formal housing structure (Nijman, 2008). The consequences of this change remain understudied in the current literature due to which the actual causes of the rebound phenomenon are not known. It is because slum rehabilitation is fairly a recent phenomenon that has been adopted by the Mumbai government, and its efficacy remains to be contested (Bardhan et al., 2015a). Most of the studies on Mumbai slums and its removal efforts focus on the income-driven factors like economic poverty as the primary cause of rebound phenomenon. Some very recent studies have recognised the potential of non-income drivers like the quality of housing, access to open spaces and the feeling of community, as defining components of occupants’ well-being in low-income houses (Nijman, 2015), (Bardhan et al., 2018c), (Vaid & Evans, 2017), (Bardhan et al., 2018d), (Sunikka-Blank, Bardhan, & Haque, 2019). Chikaraishi et al. (Chikaraishi, Jana, Bardhan, Varghese, & Fujiwara, 2017) have successfully demonstrated that in fact, income poverty is not a cause but an outcome of time poverty, which is caused by various non-income factors, that these rehabilitated people encounter. Sunikka-Blank et al. (Sunikka-Blank et al., 2019) have inferred that there is an invisible layer of non-income factors like built environment design, socio-cultural norms, and social practices that affect income poverty in the slum rehabilitation housing of Mumbai. Hence although income driven elements might seem to be the primary proponent of the rebound, while it might not be the case. Because seldom, people would leave a permanent free house in the megacity Mumbai to rebound back to the vulnerabilities of the temporary slums. In this study, we go beyond the superficial layer of income poverty and intend to investigate the influence of these non-income drivers through the theory of homeostasis that defines discomfort and distress with the broader notion of well-being in the built environment (Ortiz et al., 2017; Debnath, 2018).

Ortiz et al. (Ortiz et al., 2017), presents the conceptual framework of homeostasis concerning energy use in homes stating that ‘*energy use is a consequence of trying to attain homeostasis (comfort, neutral state, lack of stress)*’ (Ortiz et al., 2017). While doing so, they carefully describe the limitations of such a homeostatic theory and mention that this theory is based on tendencies exhibited in a residential context from the social-sciences lens (Ortiz et al., 2017). Our study builds on this limitation by assuming that occupants' *comfort, neutral state and lack of stress* is due to a balance in their economic, environmental and social dimensions of life that creates the sense of ‘locality’ in their built environment. The absence of this balance causes discomfort and distress in their lives that influences the rebound phenomenon.

According to the theory of homeostasis, discomfort and distress of an individual are due to a disbalance in their physical, psychological, social and behavioural elements of lives that contribute to better well-being by influencing attitudes, emotions, health, controls and habits (Ortiz et al., 2017). We adopt this definition of discomfort and distress and classify the indicators of physical, psychological, social and behavioural components as per the current literature (Nijman, 2015), (Zhang, 2016), (Bardhan et al., 2018b), (Bardhan et al., 2018c)– (Ortiz et al., 2017), (Sunikka-Blank et al., 2019), (Bardhan, Debnath, & Jana, 2019) such as the indicators for physical components of distress and discomfort is represented through thermal comfort, indoor air quality, health and hygiene. Similarly, for the social parts of discomfort and distress, residential energy use, income level, the feeling of safety and sense of community were used as the indicator. Also, for the behavioural and psychological components, space-cognition related to architectural design and subjective well-being were used as the critical indicators of distress and discomfort. We use the term discomfort and distress interchangeably across the social, environmental and economic domains to provide the holistic overview of comfort and lack of stress in the study area that can support our assumptions of the rebound phenomenon. These indicators were cumulatively used to explain the causes of the rebound phenomenon in the study area, based on the conceptual framework presented in section 3.

## 3 Conceptual framework

**Fig. 1:**
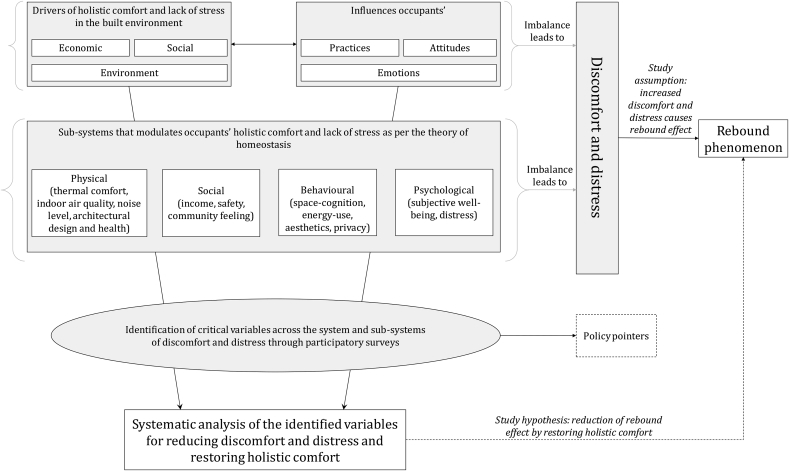
A conceptual framework based on the theory of homeostasis for deriving critical policy variables for sustainable slum rehabilitation design and planning.

A systems-of-systems approach was adopted in designing the conceptual framework for estimating the indicators of occupants' discomfort and distress in their built environment. Systems-of-systems approach refers to a multi-system organisation where a broader system is made up of several sub-systems that have inherent interdependencies within them (Hall, Otto, Hickford, Nicholls, & Tran, 2016). The conceptual framework presented here has three levels of systems and interacting sub-systems (see Fig. 1), where the drivers of holistic comfort and lack of stress are divided across three broad systems: economic, social and environmental. Imbalance in any of these systems affects their household practices, attitudes and emotions. For example, in this study for the case of slum rehabilitation housing, the rebound effect is hypothesised to be an outcome of this imbalance as occupants cannot achieve holistic comfort in their newly built environment. To retain balance regarding holistic comfort and lack of stress, occupants modulate their habits, attitudes, emotions and control to neutralise discomfort and distress across their physical, social, behavioural and psychological systems (see Fig. 1). This process of modulating one's habits, attitudes, emotions and control to regain the neutral state of lack of stress and holistic comfort is known as homeostasis (Ortiz et al., 2017).

The physical, social, behavioural and psychological systems represent the sub-systems that affect homeostasis at an individual level, significantly influencing their attitude, emotions, health, control and habits, as mentioned above. Assessment of these sub-systems can derive critical routes of assessing the causes of discomfort and distress in slum rehabilitation housing, which can be essential policymaking variables for a sustainable slum rehabilitation process. In this manner, we expand the application of the theory of homeostasis in assessing the sustainability of a slum rehabilitation process through the lens of occupants’ holistic comfort and lack of stress in their built environment.

## 4 Methodology

Household surveys were conducted to assess the determinants of discomfort and distress based on the conceptual framework illustrated in Fig. 1. The surveys were designed based on a participatory backcasting approach to understand the current state of discomfort and distress in the study area and to derive occupants-led recommendations for mitigating the loss of homeostasis. This approach gained substantial credibility as a policy modelling tool through the Dutch Sustainable Technological Development program in the 1990s, where sustainability future scenarios were developed by working backwards to address essential needs like housing, transportation and food. Its contemporary versions (called Backcasting 2.0) was further applied for policy programs in climate change (van de Kerkhof, Hisschemoller, & Spanjersberg, 2002), sustainable housing (Green & Vergragt, 2002), (Quist, Knot, Young, Green, & Vergragt, 2001), the hydrogen economy (Hisschemöller, Bode, & van de Kerkhof, 2006), for industrial coatings (Partidario & Vergragt, 2002), local and regional planning (Quist et al., 2001), (Carlsson-Kanyama, Dreborg, Moll, & Padovan, 2008) to derive policy variables concerning technical, social and cultural aspect of sustainability through participatory survey-based approaches (Paehlke, 2012). In this study, we expand the existing applicability of this participatory backcasting approach for sustainability assessment of low-income settlements like the slum rehabilitation housing programs.

**Fig. 2:**
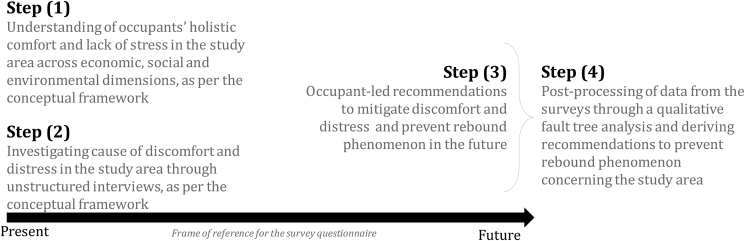
Backcasting-based study methodology.

The basic structure of our version of the participatory backcasting method consists of four steps. These steps guide the intent of the surveys; in this case, it is to investigate the causes of discomfort and distress in the study area to prevent rebound phenomenon (see Fig. 1). The four-step process backcasting-based survey methodology is illustrated in Fig. 2. The step (1) involved examination of the current drivers of occupants’ holistic comfort and lack of stress based on the economic, social and environmental sub-systems illustrated in the conceptual framework in Fig. 1.

The step (2) involved assessment of the causes of discomfort and distress along the attributes of attitudes, habits, controls and emotions to understand occupants' implications with the loss of homeostasis (as mentioned in Fig. 1). It began with broad objective questions like ‘*What would you do if the living conditions here worsen in the future?‘; ‘If the concerned authorities do not take any action in improving the SRHs, will you contribute towards community development or you move back to slums?‘; ‘What are the major causes of discomfort and distress in your current housing as compared to the horizontal slums?*’ These questions set the backcasting theme of the interviews as these questions probed probable future scenarios. Following this, specific questions were asked to understand occupants' attitude, emotions, health, control and habits of the occupants, as per Ortiz et al. (Ortiz et al., 2017) which were divided across the themes of built-environment, socio-economic, social and architectural, health and subjective well-being (see Table 1).

**Table 1:** Questionnaire variables to capture attitude, emotions, health, control and habits of the occupants (after (Bardhan et al., 2018c)).

Environmental (adapted from (Debnath et al., 2017), (Ortiz et al., 2017), (Bardhan et al., 2018d), (Nutkiewicz et al., 2018))	• Thermal comfort perception at home
	• Perception of thermal comfort as compared to horizontal slums
	• General feel at home.
	• Control means of thermal comfort at home (Mechanical/Natural/Ceiling fan only)
	• Windows and door operating schedule and its triggers
	• Perception of indoor air quality at home
	• Common diseases related to their built environment
Socio-economic	• Appliance ownership
	• Electricity use
Social and architectural (Bardhan et al., 2018d), (Sunikka-Blank et al., 2019)	• Housing design and space-related attributes like the presence of social and outdoor spaces, access to daylighting and fresh air
	• Lack of safety, social isolation, lack of privacy, noisy environment,
Health and subjective well-being (adapted from (Kern, Waters, Adler, & White, 2015))	• Happiness perception, health perception, positivity at home, satisfaction at home
	• Feeling accomplished and supportive at home.
	• Feeling valuable/worthy at home.
	• Feeling angry/joyful.
	• Feeling anxious, sad, lonely at home.
	• Feeling responsible at home.

The step (3) involved interviews concerning a future ‘*desired*’ scenario where occupants are in a balanced state of comfort and lack of stress in their built environment. This step was essential to understand occupants' expectation from their built environment and derive countermeasures to mitigate discomfort and distress in the study area. At this stage of the survey, occupants were asked about their idea of a better SRH community for their social, environmental and economic well-being. Here, it was assessed whether the social, environmental and economic discomfort and distress are compelling factors behind the rebound behaviour, or whether it is purely related to low-income and economic poverty.

The final step (4) involved post-processing of the interview data using a qualitative fault tree analysis (FTA)^1^ to represent the causes of distress and discomfort systematically and to logically list the bottom-up recommendations for reducing the rebound effect in the study area. In doing so, we address the *research question 1* through step (1) and step (2) and create a way forward for *research question 2* through step (3) and step (4) (see Fig. 2).

### 4.1 Study area

**Fig. 3:**
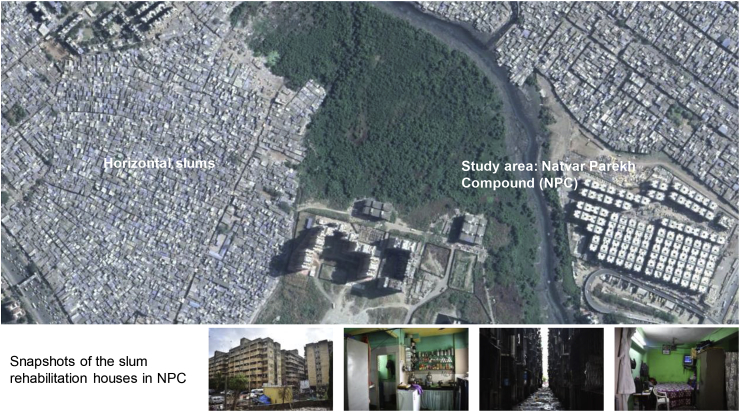
Survey area: Natvar Parekh Compound (NPC) in Mumbai, India (Map source: Google).

Thirty household surveys were conducted in the slum rehabilitation site of Natvar Parekh Compound (NPC) Mumbai, India (see Fig. 3). The NPC hosts more than 10,000 people living in 53 buildings, spanning over an area of 54,600 square metres (or 5.16 ha). Previous studies have found that the architectural structure of the buildings was too constricted (the width between each building was around 3 m) which caused a lack of air movement for natural ventilation and daylight unavailability (Bardhan et al., 2018b), (Bardhan et al., 2018c). Studies have also reported a higher occurrence of respiratory diseases, including tuberculosis in this area (Bardhan, Jana, Singh, & Pardesi, 2018e). Social issues like a higher level of alcoholism and tobacco usage with its associated anti-social effects like domestic violence are also widely reported in the existing literature (Green Health Foundation, 2016).

The surveys occupants were selected based on stratified random sampling. Only the head of the households was surveyed, in case the head was absent, the available adult household member was interviewed. The language of the interview was in Hindi or Marathi. As mentioned in the previous section, unstructured interviews were the primary medium of the surveys as it provided the flexibility of adapting the questionnaire based on the responses of Step (1), (2) and (3) of the methodology (see Fig. 2). Owing to the broad definitions of discomfort and distress, adopted in this study (see section 2.2) this format of conducting interviews were particularly crucial in capturing occupants’ attitude, emotions, health, control and habits. This format of interview allowed the respondent to talk in some depth, choosing their own words.

## 5 Results

### 5.1 Narrative based description of the survey

Narratives were collected through informal conversations using survey the execution method presented in Fig. 2. Occupants were asked on their comfort and lack of stress perceptions to record their state of homeostasis with the social, economic and environmental boundary of this study. Occupants revealed “… *we have to work indoors all the time*” which has drastically reduced their socialising time. They emphasised that “*no common private space*” is available which is a primary discomfort in living in the SRH. According to them, shifting of their activities indoors from outdoors (as compared to the horizontal slums) affected their social life. Almost every interviewee reported “*children do not have enough open spaces to play in front of our eyes … we fear they will wander off in the vertical maze*”. This lack of outdoor and social spaces for cooking and socialising is a social-distress in the SRH.

Their perception of thermal comfort is closely related to the amount they had to spend on electricity bills that closely modulated their behaviour and control of the devices. The female respondents said that they refrain from using electrical appliances for their comfort during the day due to high electric bills. They usually operate them when children and male members were present indoors, mostly during the nigh-time. This observation was also reported in the work of Sunikka-Blank et al. (Sunikka-Blank et al., 2019). While interviewees mentioned that their thermal comfort perception indoors increased as compared to the horizontal slums due to slightly bigger rooms and high-rise living, but they prefer sitting outside in the social spaces in the horizontal slums. It is interesting to observe here that occupants’ thermal neutrality is inclusive of their social-process which supports our hypothesis that change of social-process is a significant contributor towards the loss of homeostasis. Supporting quantitative data is presented in Section 5.2.

During the collapse state discussions (Step 2), most of the respondents said that even if the situation in SRH worsens, they will continue to stay because “*these problems are trivial when we have a permanent house in Mumbai*”. However, given a better house “*with more space*” to accommodate the larger household size.

While few respondents had a definite idea about an improved situation and said that cleaning the neighbourhood is very important as it could free up some spaces for social activities. Unmistakably, lack of ventilation in their living spaces was a problem for which was reported as “*not in our control because the house design restricts airflow*”. However, they were not interested in investing in the exhaust fan in fear of burdening the electric bills. They revealed that lack of daylight promoted them to switch on the lights during the day, and even cannot use the corridors for socialising as its “*too dark to even see*”. Social isolation or ‘*feel lonely and want to go back to horizontal slums*” was consistently reported as a primary cause of distress. Visiting family and neighbours were considered as “*hassle and cannot do it regularly”.*

### 5.2 Qualitative description of the survey

**Fig. 4:**
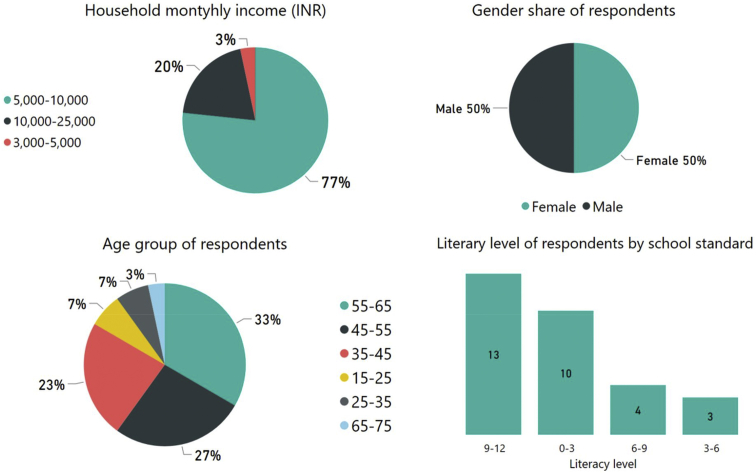
Demographic descriptive of the surveyed households.

The demographic descriptive of the survey is illustrated in Fig. 4, where the monthly income of most of the respondents was within the range of 5000–10,000 INR (80–150 USD) at 2018 rate which categorises them in the low-income population category. Every surveyed household had only one income earners. The average household size was six with a high level of illiteracy (10 out of 30 responders did not go to school). The mode age-group of the respondents was 55–65 years with an equal representation of both the gender.

#### 5.2.1 Economic attributes: appliance ownership and residential electricity-use

**Fig. 5:**
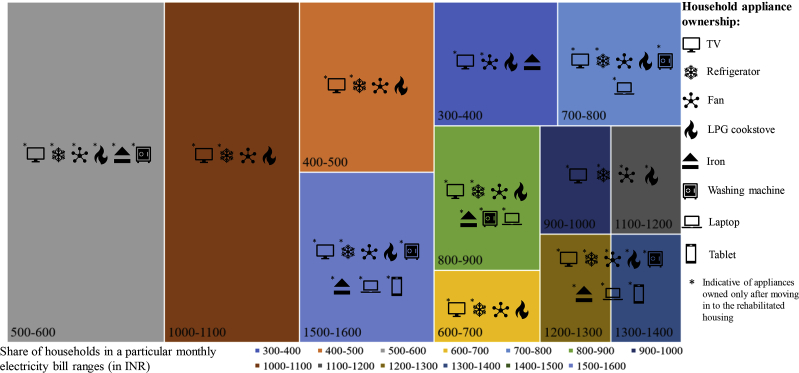
Monthly electricity bill and appliance ownership in the households.

As mentioned in section 1, low-income remains a constant source of discomfort and distress; however, investigating the non-income driver of economic distress revealed valuable information. Appliance ownership is a widely used socio-economic indicator in such low-income households, where with increase in household income the appliance ownership increases (as suggested by the energy ladder concept (Van Der Kroon, Brouwer, & Van Beukering, 2013), (Dhanaraj, Mahambare, & Munjal, 2018)). Moreover, with an increase in appliance ownership, household electricity usage also increases which increase the overall energy bills. In our surveys, we investigated whether occupants purchased more appliances on moving into the slum rehabilitation housing (SRH) from the horizontal slums. It was indeed found that respondents bought energy intensive electrical appliances, that increased their overall monthly electricity bills. Given their low-income status (see Fig. 4), this rise in electricity bills was a cause of economic distress in the surveyed households. The distribution of appliance ownership with respect to the monthly electricity bills (in INR) is illustrated in Fig. 5. It shows that the most commonly bought new electronic items on shifting to the SRH were television sets (TVs), ceiling fans and refrigerator.

Through narratives, it was found that occupants tend to purchase more appliance as they feel the fulfilment of their aspiration of ‘*having a permanent home*’. Horizontal slums were always treated as a *tempora*ry place of stay, and these SRHs gave them a permanent identity. However, this *aspiration-driven* appliance purchase increased its monthly electricity bill by 40%. It was found that in horizontal slums the average monthly electricity bill was around Rs. 300 (∼4 USD) which rose to around Rs. 500 (∼7 USD) in the study area. It was a significant economic burden on the surveyed households.

There is no discernible pattern of appliance ownership and monthly electricity bills, as it can be seen from Fig. 5 that the households with a monthly electricity bill in the range of Rs. 1200–1600 (∼13–17 USD) has almost the same number of appliance ownership as that in the lower billing range of Rs. 500–600 (∼6–7 USD). It indicates a possible tendency of the occupants in purchasing appliance out of personal *aspirations* and not out of their income-driven capabilities. The same logic applies for the group 500–600 which have more appliances than the higher billed 1000–1100 group (see Fig. 5).

The interviews also revealed that owing expensive and energy-intensive devices like a washing machine, refrigerator, computer, laptop and microwaves were considered as a significant rise in the *social-ladder* among the occupants, and they strive to buy these appliances by taking loans from friends, neighbours and informal money lenders. The burden of loan further adds to their economic distress.

Additionally, as mentioned in section 5.1, female members consistently reported of ‘*feeling lonely*’ and social isolation, interviews have revealed that this feeling of being socially isolated further leads to extended use of information and communication technology (ICT) devices like TV and smartphones for entertainment purpose. It can be interpreted than social isolation has a stronger influence than economic factor on appliance usage, as the respondents had always lived a ‘community-life’ in the slums but the vertical structures altered it to an ‘individualistic-life’.

In step (3) of the survey (see Fig. 2), most of the respondents implied towards setting up a micro-finance mechanism so that they can borrow money while purchasing appliances, instead of borrowing from their friends, relatives or neighbours. Such an institution can advise them on spending money on appliances. They wished for a community-driven microfinancing mechanism that can provide them with loans for appliances from their savings which can ease them of some economic distress. This type of mechanism is widely practised in the form of women-led self-help groups in the slums for delivering educational and healthcare loans (Sunikka-Blank et al., 2019). However, it is unlikely that the distress from the higher electricity bills can be reduced through such microfinance mechanism, but they can at least aid them in managing their finances while purchasing the appliances on moving into the SRH from slums.

#### 5.2.2 Environmental attributes

**Fig. 6:**
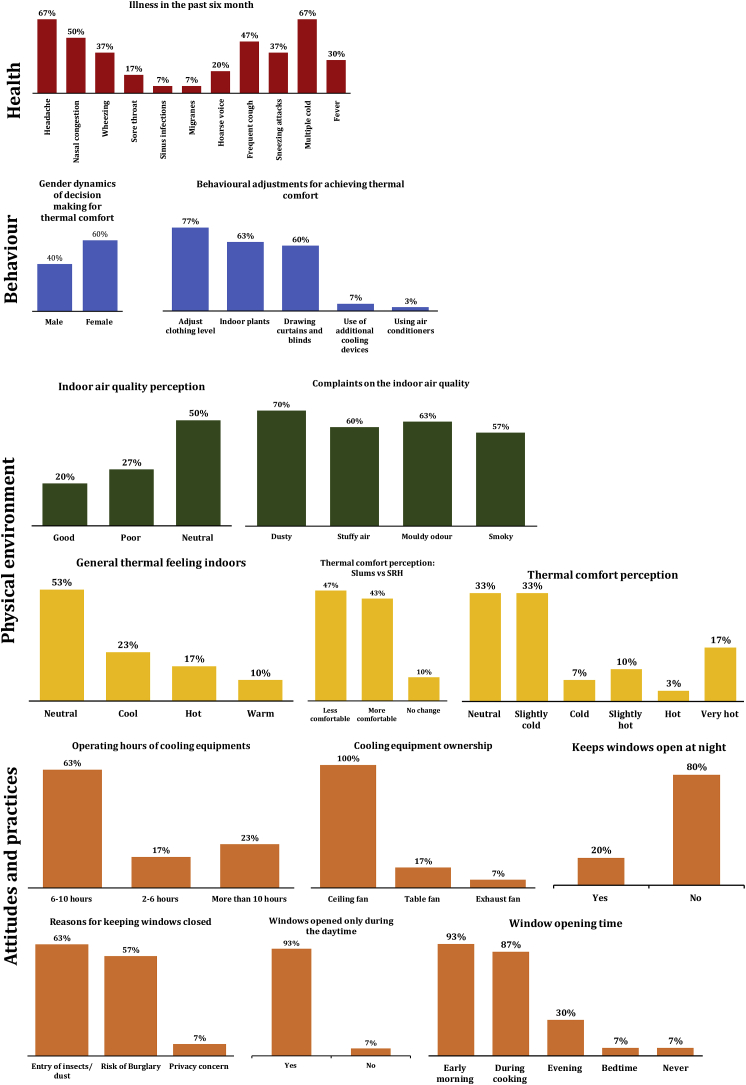
Environmental attributes that contribute to discomfort and distress in the study area (n = 30).

The general feeling regarding thermal comfort is neutral among the respondents (see Fig. 6). While enquiring about the thermal perception concerning the horizontal slums, 43% of the respondents mentioned the current houses as ‘less comfortable’ (see the physical environment in Fig. 6). Similarly, while interviewing on the perception of indoor air quality (IAQ), more than half of the respondent reported the IAQ to be poor to neutral, but only 6 out of 30 respondents said they were satisfied with the current state of IAQ and rated it as ‘good’ (see Fig. 6, physical environment). It indicates that thermal discomfort and poor IAQ were essential but not significant factors of built environment related discomfort in the surveyed households. However, respiratory diseases like headache, nasal congestion, multiple-cold, frequent cough and sinus attacks were the most commonly reported ailment in the surveyed households (see Fig. 6), which point towards poor IAQ as a significant source of distress manifested through poor health conditions. The most common complaints regarding poor IAQ were dust, stuffy air, mouldy odours and accumulation of smoke from cooking (see Fig. 6, physical environment). It was due to the lack of air exchanges through natural ventilation. The surveyed households did not have any mechanical cooling and air exchange devices. They used ceiling fans throughout the day to circulate the stale air indoors, which may be further related to the higher occurrence of respiratory diseases in the study area. It established a critical link between the quality of the built environment and health outcomes which contributes to the built-environment related distress of the surveyed occupants.

Common attitudes and practices involved keeping windows closed throughout the night-time due to the entry of insects/dust and a higher risk of burglary (see Fig. 6, attitudes and practices). The windows are generally opened during the day-time and while cooking, but during these operating hours only the female member of the households stay indoors, the male and children remain outside. It is only during the night-time all the members remain indoors, but the windows are kept closed due to the reasons mentioned above. This practice further contributes to stuffy air, thermal discomfort and lack of ventilation which relates to their built-environmental related discomfort.

The most common behavioural adjustment for mitigating thermal discomfort is by adjusting clothing levels, use of indoor plants and drawing curtain or blinds (see Fig. 6, behaviour). Female members were the decision-maker regarding thermal comfort-related controls in 18 out of 30 houses (see Fig. 6). A typical practice among the female members is to switch off the ceiling fans when the male members and children are not at home to save electricity and reduce electricity bills. It can be seen from Fig. 6 that the mean operating hours for the fan is 6–10 h, which is mostly during the night-time when everyone is indoors. However, in doing so, they experience added discomfort. It was a common practice in their horizontal slums because they used to spend most of their day-time in cool outdoor spaces, but on shifting to the vertical houses, they cannot sit outside due to lack of community spaces. Thus, the respondents revealed that lack of community space is acute distress to them and the primary cause of built environment related discomfort when compared with the horizontal slums. The step (3) of the survey (see Fig. 2) revealed that occupants (especially female participants) wanted open spaces to socialise and maintain their holistic comfort which can prevent their social isolation and thus, prevent them from spending extended time indoors watching TV. These observations enable in the drawing a causal link between built environmental related discomfort with the social distress in the fault tree (see section 5.3).

#### 5.2.3 Socio-cultural and architectural attributes

**Fig. 7:**
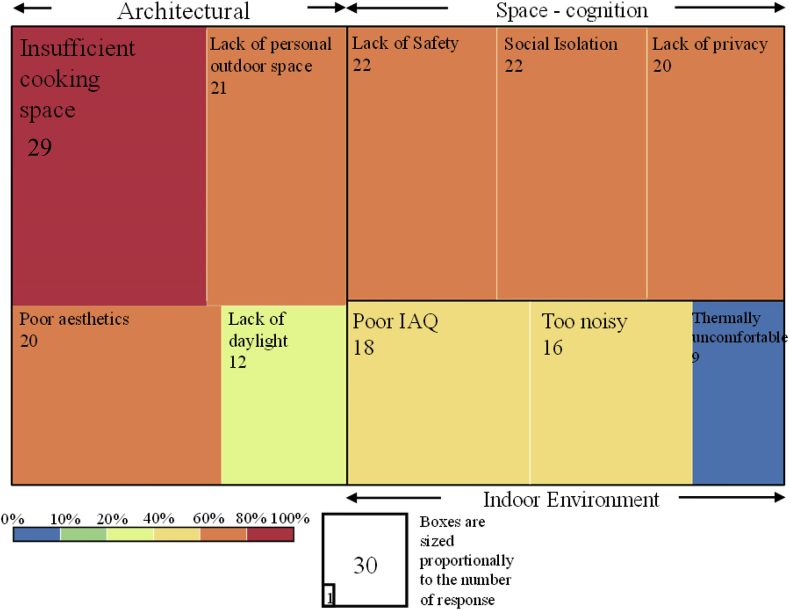
Social and architectural attributes that contribute to occupants' discomfort and distress (n = 30).

Previous research showed that nearly 70% of all residents of Dharavi slums, the most valued aspect of living is the ‘*feeling of community*’ (Nijman, 2015). This aspect was surveyed through the lens of the social and architectural attribute that contributed to their community-feeling (after (Bardhan et al., 2018d)). Informal conversations have revealed that the most common reason behind occupants' distress is lack of outdoor spaces (also mentioned in the earlier sections). It can be seen in Fig. 7 that more than 80% of the respondents are distressed about insufficient cooking spaces, lack of personal outdoor space, poor aesthetics of their surrounding along with lack of daylight in the living space. Higher noise levels due to their proximity to industrial zones and highways was also a critical distress factor (see Fig. 7). These architectural elements are an essential contributor to their loss of homeostasis and form a vital part of the fault tree (see section 5.3).

Additionally, as discussed earlier in section 5.1, social isolation is a significant cause of distress to the female respondents because they could not perform their typical social processes in the NPC. Apart from it, lack of safety and daylight availability in the corridors and in between the buildings makes it difficult for them to socialise even with their neighbours and is a significant cause behind their discomfort in their built environment (see Fig. 7). Occupants also revealed that to cope with this social isolation, they often keep the TV in ON-state in the background. It further adds to their increased electricity bill, which adds to both economic and built environment related distress (also mentioned in the economic attribute of section 5.1). Thus, it further strengthens the causal link between built environment discomfort and economic discomfort with social distress related to lack of community spaces in the qualitative fault tree (see section 5.3).

During the step (3) of the survey, (see Fig. 2) occupants said that in a desirable future they would want their available open spaces to be kept clean and well-maintained so that it can be used as social spaces. They revealed that the people in the locality are keen on maintaining the open spaces provided they are paid regularly to do that job. It can be done by forming a ‘cleaning committee’ which can aid in keeping the available open spaces clean, safe and hygienic for everyone to use as socialising spots.

#### 5.2.4 Subjective well-being of the occupants

As a part of the homeostatic theory, the well-being of the occupants was investigated. The multivariate PERMA methodology of Kern et al., 2015, was adopted for this investigation and responses were scored on a Likert like scale of 0–10. A score of 0 indicated the least satisfaction, whereas a score of 10 reported the highest rate of satisfaction. Table 2 illustrates the mean and mode scores of the PERMA indices. Responses show that the surveyed occupants were satisfied with their household responsibilities and its sense of accomplishment regularly. They feel that they contribute significantly to the progress of their households (HH) (Mean = 7.90, Mode = 9), which make them happy (Mean = 7.26, Mode = 8) and appreciated by the family members (Mean = 7.66, Mode = 10). They believed that they are in sound health (Mean = 7.96, Mode = 8) and were satisfied with the current state of health (Mean = 7.66, Mode = 8) (see Table 2).

**Table 2:** Descriptive of respondents’ subjective well-being based on the questionnaire of Kern et al. (Kern et al., 2015).

Parameters	Mode score (out of 10)	Mean score (out of 10)
Perception on household goals and responsibilities		
Making progress in the household	9	7.90
Satisfaction with the household chores	8	7.76
Happiness in the household	8	7.26
Appreciation by the family members	10	7.66
Family support	10	7.23
Value in doing household chores	10	7.46
Interest in doing household chores	8	7.36
Sense of direction in the household	6	7.36
Feeling of goals achieved in the household	9	7.83
Handling household related responsibilities	10	7.70
Satisfied with the family relationships	10	7.90
Satisfied with work	8	6.66
Perception of their personal health		
Personal health rating	8	7.96
Satisfaction with current health status	8	7.66
Health status compared with neighbours	8	8.00
General feeling and emotions in the built environment		
Feeling positive	7	6.56
Feeling angry	0	3.90
Feeling joyful	9	8.30
Feeling anxious	0	3.66
Feeling sad	6	4.93
Feeling contended	10	7.53
Feeling lonely	1	4.00
Feeling focussed	0	4.60

The interviewees scored higher on positive well-being indices like feeling joyful (Mean = 8.3, Mode = 9), contended (Mean = 7.53, Mode = 10), and while scored significantly low when asked about their general focussed-at-work (Mean = 4.6, Mode = 0) (see Table 2). When asked about their feeling of being positive with the current living conditions, they reported mid-range scores (Mean = 6.56, Mode = 7) along with being sad (Mean = 4.93, Mode = 6) in the rehabilitated housing. They felt lonely in the NPC (Mean = 4, Mode = 1), due to lack of *community-feeling* and it was the major cause of psychological distress among the occupants.

The negative emotions like ‘*feeling lonely*’, ‘*feeling anxious*’ and ‘*feeling angry*’ were inversely scored, i.e., lower scores on these feelings indicate higher weight. For example, in Table 2, the mean score for feeling lonely is 4 out of 10 whereas the mode score is 0 out of 10, it indicates that most of the surveyed occupants responded that they *feel very lonely*, as ‘0 = very lonely’ and ‘10 = not lonely at all’. Similarly, for *feeling anxious*, the mode score is 0, whereas the mean score is 3.6 indicating a higher occurrence of anxiety among the surveyed occupants. Occupants have scored feeling angry with a mean value of 3.9 and a mode value of 0 (see Table 2), indicating the dominance of these three negative emotions among the surveyed units.

Female respondents reported even lower score on the feeling of loneliness (See Table 3) (mean = 2.6, Mode = 0), as females spend most of their time in the home while the working male of the household goes out of the house to do their jobs. As discussed in the earlier sections, in horizontal slums, these female members used to spend most of their time in outdoor spaces, whereas in the vertical structure of the NPC they are detached from such community engagements. It further relates to the proposition of providing social-spaces to improve their positive and negative emotions, as stated by the occupants in the step (3) of the survey (see Fig. 2).

**Table 3:** Descriptive of negative feeling of surveyed respondents.

	Gender	Feeling angry	Feeling anxious	Feeling lonely
Mean (out of 10)	Male	5.26	4.60	5.40
	Female	2.53	2.73	2.60
Mode (out of 10)	Male	5	5	0
	Female	8	8	6

[Note: The scores are inversely related to the weights, i.e., lower scores have high weightage].

### 5.3 Qualitative fault tree

**Fig. 8:**
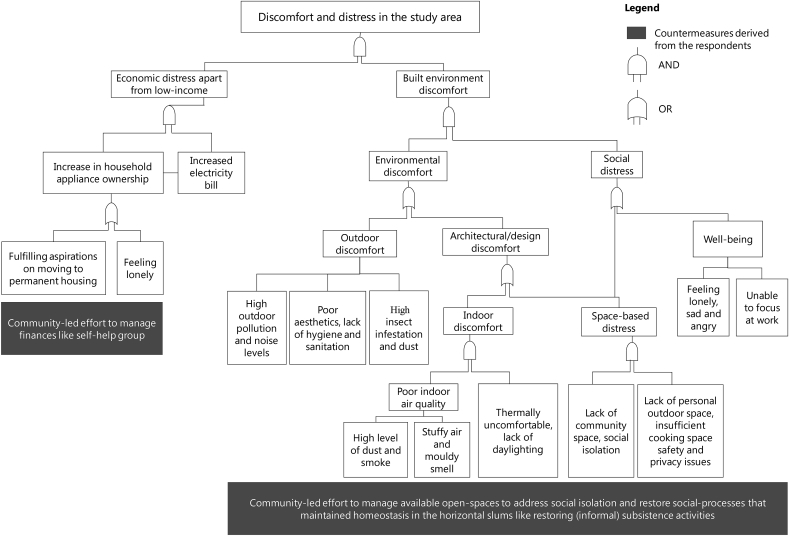
Fault tree indicating the determinants of discomfort and distress in the study area. [Note: Low household income remains constant economic distress and we have not explicitly mentioned it to highlight other non-income drivers].

Based on the evidence gathered in section 5.1, 5.2, a qualitative fault tree was constructed as a part of step (4) of the study methodology (see Fig. 2). The qualitative fault tree is illustrated in Fig. 8 that indicates two distinct causes of distress and discomfort in the study area. Economic distress is caused due to higher electricity bills owing to higher appliance ownership by the low-income surveyed households, the evidence of it is presented in the ‘economic attributes’ segment of section 5.2. It is also related to occupants' social isolation, which is manifested through poor built environment design of the study area (see Fig. 8). Economic distress is also found to be related to the aspirational aspect of the surveyed occupants. These factors act as non-income drivers of economic distress in the study area, as poverty remains a constant source of distress for every surveyed occupant.

Similarly, the built environment discomfort is explained through two branches that categorise environmental discomfort and social distress which are discussed in detail in the environmental attribute, social and architectural attribute and the subjective well-being segments of section 5.2 (see Figs. 6 and 7 and Table 2). The critical component of environmental discomfort is through the poor design and planning of the studied SRH that causes poor indoor air quality, thermal discomfort, lack of daylighting in the living spaces and high level of noises (see Figs. 8 and 7). Besides, another causal connection is established between space-based distress owing to lack of community spaces that cause social isolation among the surveyed occupants (especially among the female members) (see Fig. 8). It also links the distress caused due to lack of personal outdoor spaces and cooking space, along with inadequate safety and hygiene (see Figs. 8 and 7). The social distress is further revealed through poor subjective well-being concerning loneliness and unable to focus at work (see Fig. 8, Table 2, Table 3).

The constructed fault tree also reveals the recommended mitigation (see Fig. 8) measures of discomfort and distress. These counter-measures were derived during the step (3) of the survey (see Fig. 2) through the unstructured interviews and are entirely based on occupants’ recommendations. Based on the assumptions of this study, it is expected that these recommendations can aid in mitigating discomfort and distress in the study area and contribute towards reducing the rebound phenomenon.

## 6 Discussion

In this study, a conceptual framework was presented to investigate a rebound phenomenon in the slum rehabilitation of Mumbai from the lens of occupants' discomfort and distress in their built environment (see Fig. 1). The rebound phenomenon is assumed to be a result of heightened discomfort and distress among the occupants who were shifted to the slum rehabilitation housing from horizontal slums. The conceptual framework was based on the notion of holistic comfort and lack of stress after Ortiz et al. (Ortiz et al., 2017) that consisted of social, environmental and economic dynamics of occupant's life that contribute to their better well-being. Household surveys were conducted across these broad domains that revealed multidimensional causes of discomfort and distress which are critical in reducing the rebound phenomenon and ultimately improving the sustainability of the slum rehabilitation process (see Fig. 1).

Results were collated through a qualitative fault tree analysis, illustrated in Fig. 8 which shows that discomfort and distress in the study area can be categorised in two broad domains. Economic distress is caused continuously due to the low income of the surveyed occupants, but higher electricity bills also contributed to it (see Fig. 8 and ‘economic attribute’ of section 5.2). Even though the socio-economic status of the occupants was similar (77% earned less than 10,000 INR (∼150 USD) per month), the appliance ownership varied (see Fig. 5). The purchase of appliances was primarily to mitigate well-being related distress like social isolation. Television was the most common appliance in all the households, which is a change from that of slum habitats, where watching TV was a group activity. Hence, television and ICT devices act as de-stressing appliances in the SRH which contributed to the neutrality of comfort and lack of stress.

Interestingly, the number of appliances was not linearly related to the electricity bill or income which in general should be the case (see Fig. 5). To achieve holistic comfort, the occupants were paying 10–28% of their income for paying electricity bills alone, which itself is alarming given that SRH gets special electricity subsidy (World Bank, 2008). Almost 83–90% of the surveyed occupants reported that they resort to cooling devices only when male members or guests are in the house (see Fig. 6). It shows that the socio-cultural dynamics significantly burden the social, environmental and economic comfort and lack of stress conditions even though that lack of daylight and stuffy air (i.e. poor ventilation) were the major indoor environmental concerns of the occupants in SRH (see Figs. 7 and 8). It further strengthens our proposed conceptual framework on having a broad definition of comfort and lack of stress for improving the sustainability of the slum rehabilitation process (see section 3 and Fig. 1).

In terms of discomfort and distress associated with the built environment, the qualitative fault tree reveals important interdependent linkages between environmental discomfort and social distress (see Fig. 8). The surveyed occupants were leveraging one discomfort/distress to mitigate another, which resulted in heightened discomfort and distress. For example, 14 out of 30 respondents reported “less thermally comfortable” in comparison to slums, most of the occupants operated the ceiling fan for cooling only for 6–10 h daily (see Fig. 6). It depicts the discomfort that the occupants mitigate by extending their energy budget leading to economic distress in the form of electricity bills (as illustrated in the fault tree in Fig. 8).

The complaint regarding the amount of space for cooking was a direct indication of the change of practice in cooking in comparison to the slums (see Fig. 7). Numerically, the space for cooking provided in the multipurpose room is enough (Debnath, Bardhan, & Banerjee, 2016), the practice of cooking in open space in the slums was a sharp contrast to the provided indoor cooking space. The loss of homeostasis due to a change of practice prompted by the built environment is an inimitable finding and needs to be addressed through policy (also reported in (Sunikka-Blank et al., 2019)). It supports our initial assumptions for the conceptual framework in Section 3 (see Fig. 1).

It is also interesting to note that while most of the occupants were highly satisfied with the general condition of SRH, they reported emotional distress like feeling lonely, angry and anxious (see Table 2). The high satisfaction from living in resource-constrained SRH infers that they derive satisfaction from other aspects of life by potentially offsetting the dissatisfaction occasioned by low-incomes (Biswas-Diener & Diener, 2001). However, when contrasted with their lives in slums, most of them felt social isolation and reported ‘feeling lonely’ as significant distress (see Table 2, Table 3). It further related to their overall discomfort in their built environment, as illustrated in Fig. 8.

Notably, women felt more socially deprived than men, causing a significant loss of homeostasis in comparison to the slums. In slums, the open spaces were used by women for social consumption, as they performed most of the household activities outdoors while socialising with their neighbours. This loss of access to open spaces coupled with the societal restriction of women's movement coded through hegemonic femininity or the cultural guards pushes the women to more discomfort and distress (loss of homeostasis) in such low-income settlements (Vaid & Evans, 2017), (Sunikka-Blank et al., 2019), (Ranade, 2007).

### 6.1 Recommendations derived from the community through our surveys

The occupant-led recommendations are presented in the fault tree illustration in Fig. 8. It was a part of our backcasting methodology (see step (3), Fig. 2) to derive recommendations from the occupants because the entire slum rehabilitation process, in general, is a co-operative based model between slum dwellers and the developers (see appendix). We combine these recommendations to represent one viable model of community development for the people living in the study area. The primary idea behind this recommendation is to provide a proof-of-concept of our conceptual framework in Fig. 1. The conceptual framework was designed with the intention of developing a sustainability assessment model of the current slum rehabilitation programs from the lens of the theory of homeostasis, such that the discomfort and distress can be mitigated to reduce the rebound phenomenon.

**Fig. 9:**
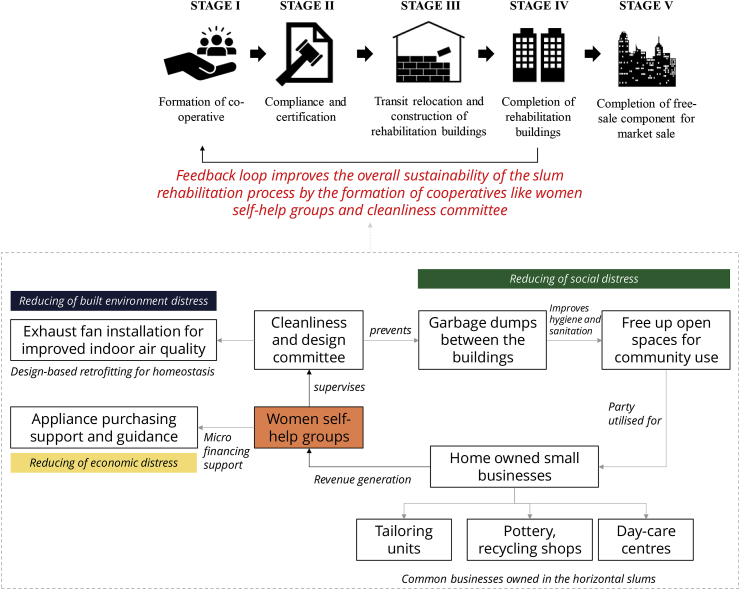
Women led self-help group model for mitigating the loss of homeostasis in the study area.

Most of the occupants, and especially female suggested community-based microfinancing and neighbourhood maintenance co-operatives as they improve accountability of the occupants in improving their built environment. Thus, enhancing their homeostasis (i.e. holistic comfort and lack of stress) in the SRH. The female respondents said that they spend most of their time indoors and engaging in a self-help group would connect them for subsistence activities and generate income. We build on these suggestions to propose a possible community-led co-operative based model for the Natvar Parekh Compound (NPC), shown in Fig. 9.

We mention a woman-led self-help group (SHG) driven cooperative model (see Fig. 9) for mitigating distress and discomfort in the study area. The organisation of the cooperative shows that the women-led SHG acts as the central governing body that operates a ‘cleanliness and design’ committee. The role of this committee is to maintain sanitation and hygiene of the open spaces. These clean open spaces will create community-owned zones and provide much-needed social spaces. It also provides the space for performing subsistence activities in the form of household businesses like tailoring shops, pottery barn, recycling collection shops, pickle making shops and children day care centre. Such businesses were familiar in horizontal slums which formed a critical part of their socio-economic life (Nijman, 2015). It will help them to mitigate social distress and restore balance in their holistic comfort (as per the conceptual framework in Fig. 1).

In return, the part of the revenue generated through these subsistence activities can be used to fund the SHG and create a micro-financing body. It can further provide the needed personal loans for buying a household appliance, without the loan-driven economic distress (as discussed in section 5.2). However, higher appliance ownership and the subsequent increase in energy intensity will increase their electricity bills. It is inevitable, and part of the development process, but household finances can be managed from the subsistence activities that can generate extra income by utilising the maintained social spaces for performing the communal activities like the horizontal slums.

The involvement of women in such community-led maintenance and cleanliness cooperatives will address their social distress concerning social isolation (see Fig. 8) and can provide them with a decision-making capability to negotiate with developers during the first step of co-operative formation phase in the slum rehabilitation process (see appendix). An initiative like this can induce a feedback loop in the current SRH process (see Fig. 9), which can have a snowballing effect on sustainable planning and design of such housing societies and can influence the reduction of the rebound phenomenon. It can fill the policy gap of missed accountability in the current slum rehabilitation policies.

## 7 Conclusion

This study forwards an innovative methodology for planning sustainable low-income settlements like the slum rehabilitation housing (SRH). The need was identified based on the current pattern of occupants' behaviour in the SRHs in Mumbai, where they are abandoning these vertical houses and moving back to the horizontal slums, presenting a rebound effect from these state-provided houses. The low-income and poverty remain a critical source of distress among the rehabilitated occupants, but there were underlying causal factors for such a rebound phenomenon. Therefore, we adopted an occupant driven investigation pathway for examining the multi-layered causes of the rebound effect of the SRH in Mumbai. The methodology consisted of two key segments: first was the conceptualisation of a bottom-up sustainability assessment method that centred around the notion of homeostasis, i.e. holistic comfort and lack of stress in the built environment. The second was the planning and design of a field survey based on the principle of participatory backcasting technique to record the dynamism of occupants’ attitudes, emotions, control, health and habits in their transitioning built environment.

It was found that the interconnected causes of discomfort and distress in the study area were due to two primary reasons, first was due to economic distress owing to low-income status coupled with higher appliance ownership and usage implicating higher monthly electricity bills. The second reason was due to poor design and planning of the SRH that altered occupants’ social structure from a community-living to an individualistic life. The poor design of the SRH prevented the respondents from using the social spaces which had changed their household practices, attitudes and emotions. These findings act a proof-of-concept for our proposed sustainability assessment framework based on the theory of homeostasis (i.e. holistic comfort and lack of stress).

In doing so, we expanded the application of the theory of homeostasis in sustainability assessment and policymaking of low-income settlements where understanding the local socio-cultural context becomes critical for the success of the project. There are three key contributions of this study, first is setting up the scope for non-income drivers of discomfort and distress in low-income housing like the slum rehabilitation housing in Mumbai that can influence the occupants to move out of such house to horizontal slums. The current literature on sustainable slum rehabilitation policies is heavily centred around ‘low-income’ specific dialogues where poverty is identified as the pivotal cause of failures. This study opens the discourse towards the causal factors that remain invisible in current literature. The proposed methodology contributes to the perspective development on sustainability and slum upgradation through an occupant-led policy development route. It provides a structure to the current bottom-up approach in slum rehabilitation policy. Critically this study contributes towards avenues of incorporating context of space in policy making rather than impulsively adopting the approach of verticalization and the control of sprawl.

The second contribution of this paper is towards the development of a reproducible and defensible method that can broadly collect and interpret causes of occupants' discomfort and distress in transitioning low-income built environment. It fills an important literature gap in low-income sustainability planning of the Global South as most of the planning methods are often borrowed and reiterated from the Global North which misses the granular aspect of socio-cultural dynamics of occupants living in poverty. The theoretical framework presented in this study can be further developed into a web-based sustainability assessment tool that can provide valuable information concerning occupants’ socio-economic and environmental needs for better well-being in low-income housing sites. It can prevent similar rebound effects of the future affordable and low-income building stocks that Global South will accommodate over the economic growth phase.

The third contribution of this study is on the development of the backcasting methodology for sustainable development of the slum rehabilitation houses. This study demonstrated for the first time that working backwards can reveal granular details on the cause of occupants’ distress and discomfort in their built environment. We anticipate that involving such backcasting methodologies in the broader policy-making framework of urban poverty alleviation will improve the robustness of the policies by introducing a feedback loop in the linear policy frameworks like the slum rehabilitation process in Mumbai. The introduction of such feedback loops and their inclusion in the current policy framework can amend the informality persistent in the Indian planning and planning institutions by improving the accountability of stakeholders from policy making to the delivery process.

This study also recognised that investigating the well-being of such low-income settlements based on a standardised set of indicators, like PERMA method, reveal counter-intuitive results. Well-being has always been a developed country notion, and it cannot be adequately investigated with those indices for the cities of the Global South. A newer method is needed to understand and explore the notion of well-being in such complex urban systems where built environmental and socio-economic factors dominate the well-being of an individual. Discomfort and distress assessment-based methods like the theory of homeostasis can fill in this gap, provided it is developed with significant empirical evidence from a multi-disciplinary perspective.

This study is limited by its small sample size, and the findings are restricted to the socio-cultural context of the study area (NPC). However, the results can be considered as a representation of the rehabilitated population in Mumbai as SRH is restricted to a similar socio-economic class which shows the possible generalisability of the conceptual framework. The methodology remains replicable and reproducible to other slum rehabilitation sites as well. The assumption of distress and discomfort causing rebound phenomenon is limited by our attribute selection which can be further refined through a larger sample size across the slum rehabilitation sites in India and the Global South. The theory of homeostasis needs to be tested and developed over a large population set, which remains a significant gap in the current literature.

## Figures and Tables

**Figure:**
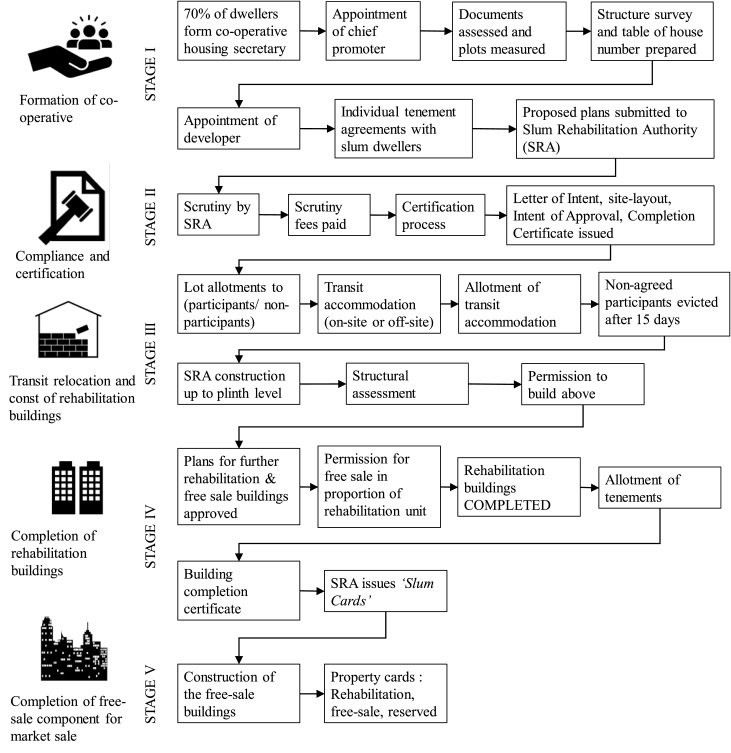


## Appendix

A1. Slum rehabilitation process flow in Mumbai, India (Source: Author's).

## Footnotes

1 A fault tree is synthesised in lines of Fault Tree Analysis (FTA). FTA is a deductive failure-based approach often used in mechanical and aerospace systems design. It starts with an undesired event (collapse scenario), followed by determining its causes by working backwards (Stamatelatos, Vesely, Dugan, Fragola, & Railsback, 2002). As a result, FTA clarifies the cause-effect relationship between the undesirable event and its causal pathways (Stamatelatos et al., 2002). Kishita et al. (Kishita et al., 2017) have used qualitative FTA was used in determining the resilience of Japan's energy system under external complexities using a backcasting approach.
